# Herpes Zoster Risk After Total Knee Replacement: a multicenter, propensity-score-matched cohort study in the United States

**DOI:** 10.7150/ijms.97654

**Published:** 2024-08-19

**Authors:** Wen-Chieh Liao, Shao-Wei Lo, Chih-Lung Wu, Sin-Ei Juang, Hui-Chin Chang, Shuo-Yan Gau, Chen-Pi Li

**Affiliations:** 1 Department of Post-Baccalaureate Medicine, College of Medicine, National Chung Hsing University, Taichung, Taiwan.; 2 Doctoral Program in Tissue Engineering and Regenerative Medicine, College of Medicine, National Chung Hsing University, Taichung, Taiwan.; 3 Harvard T.H. Chan School of Public Health, Boston, MA, USA.; 4 Department of Orthopedic Surgery, Chung Shan Medical University Hospital, Taichung, Taiwan.; 5 School of Medicine, Chung Shan Medical University, Taichung, Taiwan.; 6 Department of Anesthesiology, Kaohsiung Chang Gung Memorial Hospital and Chang Gung University College of Medicine, Kaohsiung, Taiwan.; 7 Evidence-based Medicine Center, Chung Shan Medical University Hospital, Taichung, Taiwan.; 8 Library, Chung Shan Medical University Hospital, Taichung, Taiwan.; 9 Department of Nursing & Tungs' Taichung MetroHarbor Hospital, Taiwan.; 10 Department and Graduate Institute of Business Administration, National Taiwan University, Taipei, Taiwan.; 11 Department of Pharmacology, Chung Shan Medical University, Taichung, Taiwan.; 12 Orthopedics Department, Chi-Mei Medical Center, Tainan, Taiwan.

**Keywords:** osteoarthritis, total knee replacement, herpes zoster, risk, cohort study, electronic medical records

## Abstract

**Background:** Total knee replacement (TKR) is a common surgical procedure for osteoarthritis (OA) patients. TKR may increase susceptibility to herpes zoster (HZ) by inducing immunosuppression, surgical stress, and nerve injury. However, limited data exist on the relationship between TKR and HZ. This study examined the risk of HZ over time among OA patients who underwent TKR and those who did not, using a large population-based cohort.

**Method:** Utilizing the TriNetX research network, people with OA and underwent TKR were recruited as case group. After 1:1 propensity score matching, OA patients who never experienced TKR were included as control group. Covariates, including demographics, comorbidities, and laboratory data, were balanced using propensity score matching. A 5-year follow-up assessed the hazard ratio of incident HZ and related complications.

**Results:** Compared to the control group, a significantly elevated risk of HZ was observed in the TKR cohort across 5-year follow-up period, with the hazard ratio of 1.223 (95% CI: 1.089-1.373). Zoster without complications presented 1.173-fold risk in TKR patients while comparing with non-TKR controls. However, most other secondary outcomes related to HZ complications—such as encephalitis, neurological involvement, ocular disease, and disseminated zoster—did not show a significant increase in risk. The risk of HZ was statistically significant for females and older adults in the TKR cohort than in the control cohort.

**Conclusions:** OA patients who underwent TKR had an increased risk of HZ compared to those who did not receive the procedure, especially females and older adults. These findings highlight the need for HZ monitoring/prevention protocols and further research on mitigating viral reactivation after major joint surgery.

## Introduction

Herpes zoster (HZ), a painful and debilitating neurological syndrome commonly referred to as shingles, arises from the reactivation of the varicella-zoster virus (VZV)^1^. HZ affects approximately 1 in 3 people during their lifetime^2^. The risk of developing HZ increases sharply after 50 years of age^3^. Once reactivated, the virus causes a localized, painful skin rash and blisters along dermatomal distributions^4^. HZ is associated with several acute and chronic complications, including postherpetic neuralgia, vision loss, neurological injuries, and secondary bacterial infections^5^, ^6^. Given this considerable disease burden, identifying and mitigating risk factors for HZ reactivation are crucial public health priorities.

The reactivation of VZV can be either spontaneous (70% of cases) or provoked (30% of cases) by factors that impair cellular immunity, such as cancer, HIV, steroid intake, diabetes, and tuberculosis^6^, ^7^. Emerging evidence indicates certain surgical procedures, such as dental, facial, skull, and video-assisted thoracic surgeries, may increase susceptibility to HZ postoperatively^8^-^10^. Total knee replacement (TKR) is a common orthopedic surgery that involves replacing the damaged knee joint with an artificial one. TKR is performed to restore the mobility and comfort in patients with severe osteoarthritis (OA)^11^. In the US alone, more than 600,000 TKR procedures are done every year^12^. However, there is a possibility that TKR may increase the risk of HZ, as suggested by a few case reports^13^, ^14^. Wei et. al. reported an 88-year-old woman who developed HZ on the right knee and above, three weeks after TKR^13^. Jain et. al. reported a 67-year-old woman who developed HZ in the L2-3 dermatomal area, four weeks after TKR^14^. Despite these anecdotal case reports, no large-scale studies have systematically examined the relationship between TKR and HZ.

In this study, we aimed to address this critical evidence gap by examining the association between TKR and subsequent HZ risk using a robust matched cohort design. Our goal was to determine if TKR impacts HZ risk, facilitating enhanced prevention, monitoring, and treatment strategies in this large and growing population of TKR recipients.

## Methods and Materials

### Study population

This study was performed in a retrospective cohort design. The TriNetX research network and its subset, the US collaborative network, was utilized. TriNetX research networks provide users de-identified electronic medical records from their health collaborative organizations (HCOs) for analytical purposes. This global-federated research network contains more than 120 HCOs in the system and had been utilized in large-scale studies delivering outcome evaluation^15^-^18^. In the main analysis, we utilized the US collaborative network, a subset containing more than 59 HCOs solely in the United States. This study adhered to the STROBE guideline and the need of ethical approval was exempted by the Institutional Review Board of Tungs' Taichung MetroHarbor Hospital (IRB TTMHH No.: 112207N).

### Settings and outcome evaluation

Enrollment of participants spanned by January 1^st^, 2005 to December 31^st^, 2017. All participants had a follow-up time for greater than 5 years. People with visiting record in the TriNetX system and was diagnosed of OA were included as study population in this study. All individuals less than 18-year-old, having previous cancer record and having previous herpes zoster history were excluded from further analyses. People with OA and underwent TKR within the enrollment period were recruited as the TKR cohort. To determine non-TKR control cohort, people with OA and never underwent TKR surgery were included. In the main analysis, propensity score matching was performed based on 1:1 ratio and matching covariates included potential confounders including age, sex, socioeconomic statuses, medical utilization status and comorbidities. Primary outcome was set as incident herpes zoster. Secondary outcome was set as subgroups of herpes zoster, including zoster encephalitis, zoster with other nervous systemic involvement, zoster ocular disease, disseminated zoster, zoster with other complications and zoster without complications. Information of the applied algorithms of study population, covariates and outcomes were reported in **Table S1**. To validate the results, sensitivity analyses based on different matching covariates, wash-out periods and follow-up periods and stratification analyses based on variation on age and sex subgroups were performed. For sensitivity analyses based on different matching covariates, 3 different models were applied as the following design. Model 1: propensity score matching performed on age at index, sex, race and comorbidities. Model 2: propensity score matching performed on age at index, sex, race, body mass index, lab data, status of comorbidities, lifestyle, socioeconomic issues, medical utilization status, vaccination for herpes zoster status and comedication status. Model 3: propensity score matching performed on age at index, sex, race, body mass index, lab data, status of comorbidities, lifestyle, socioeconomic issues, medical utilization status, vaccination for herpes zoster status in different definition (Zostavax® Merck, Shingrix GSK.; CPT code: 90736, 90750). For sensitivity analyses based on different wash-out periods, incident herpes zoster occurred within the period were not calculated as outcome events. In the respective 3 models, 12 months, 24 months and 36 months, were respectively applied as wash-out periods to address potential reversed causation bias. For sensitivity analyses based on different follow-up period, the follow-up-period of 8 years, 10 years and 15 years were applied.

### Statistical analyses

The TriNetX platform's analytical instruments were employed to conduct the data analysis. Difference in baseline characteristics were evaluated using standardized difference (SD). A SD value exceeding 0.1 was deemed to have significant difference. The likelihood of future herpes zoster development was assessed by calculating hazard ratios. To ascertain statistical relevance, 95% confidence intervals were determined in conjunction with the hazard ratios.

## Results

### Baseline characteristics of the study subjects

**Figure 1:**
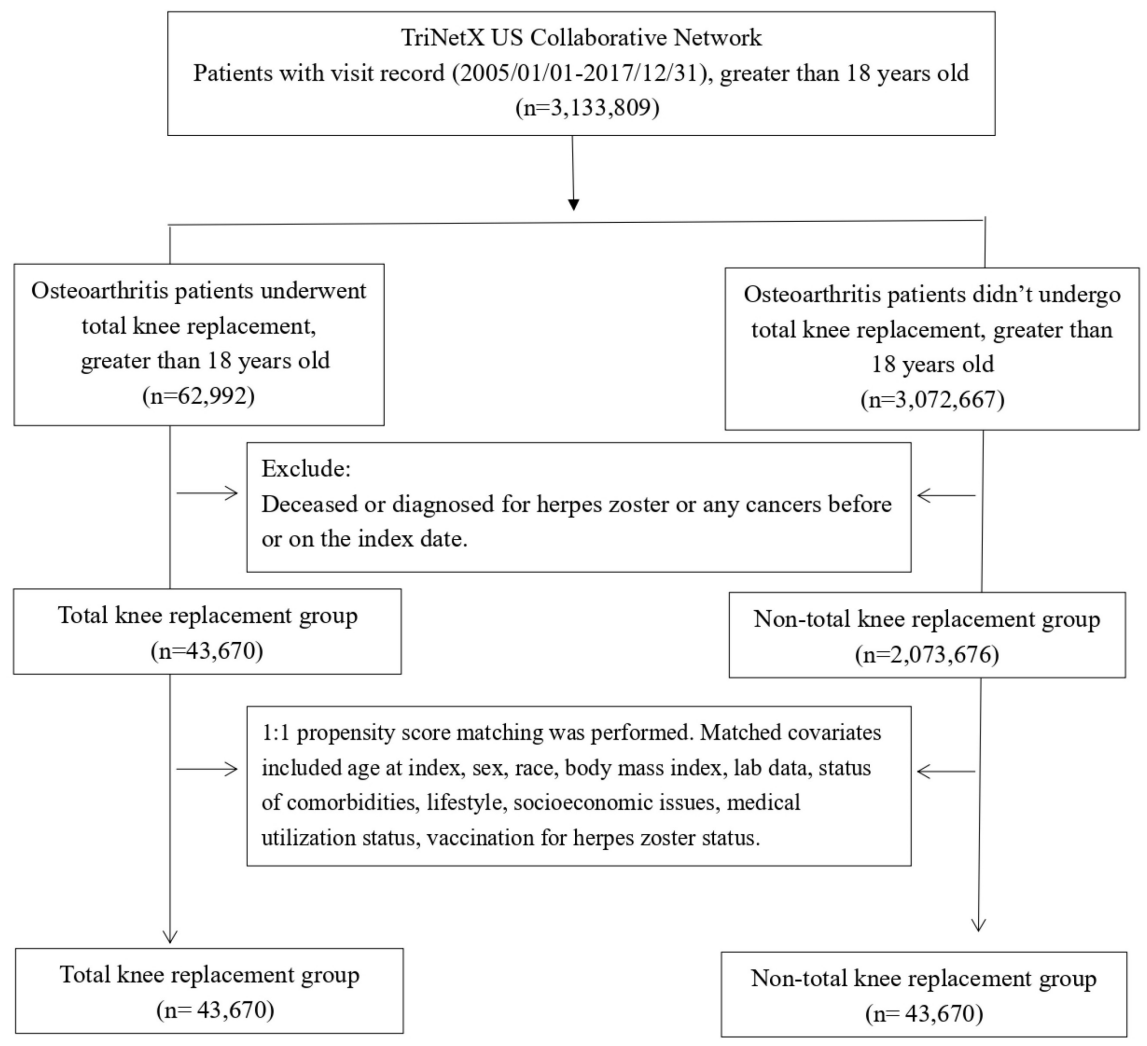
Patient selection process.

We analyzed the data from 43,670 OA patients who underwent TKR and an equal number of patients who never received the procedure (**Figure 1**). The demographic characteristics, comorbidities, and laboratory data of the two cohorts before and after propensity score matching are presented in **Table 1**. The TKR and control cohorts had similar age, sex, race, lifestyle, comorbidities, socioeconomic status, comedication status, medical utilization status, vaccination status, and laboratory data after matching, indicating a good balance between the groups (Standardized difference < 0.1). The mean age of the matched participants was 64.3±10.4 years for the TKR cohort and 64.3±10.8 years for the control cohort. Approximately 60% of the patients in both cohorts were women. The majority of the patients were Caucasian (71.8% vs. 71.9% in TKR and control cohort, respectively).

### Risk of herpes zoster after total knee replacement

**Figure 2:**
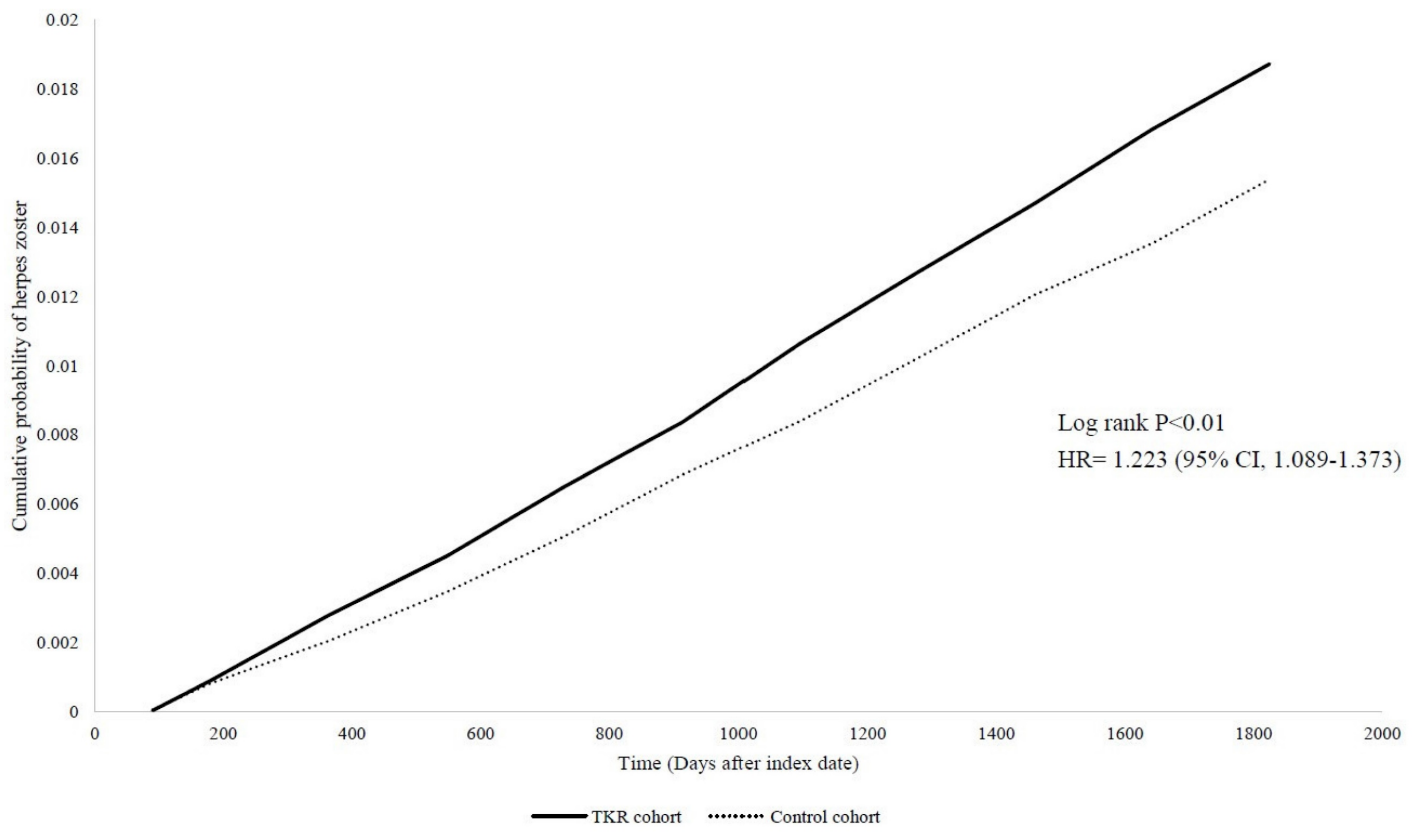
Kaplan-Meier plot of the risk of herpes zoster.

**Figure 3:**
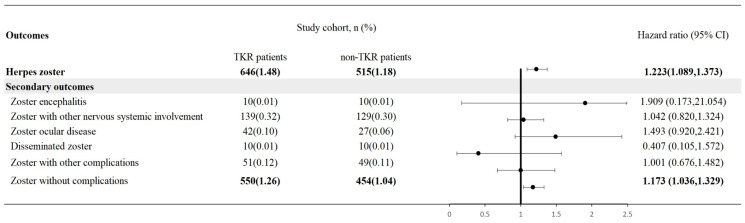
Risk of herpes zoster and secondary outcomes in people underwent total knee replacement. Legends: Data present here were the value of follow up from 90 days after index date to the respective following up years. Propensity score matching was performed on age at index, sex, race, body mass index, lab data, status of comorbidities, lifestyle, socioeconomic issues, medical utilization status, vaccination for herpes zoster status.

The risk of HZ was assessed in both TKR and control cohorts. Compared to the control group, a significantly elevated risk of HZ was observed in the TKR cohort across 5-year follow-up period, with the hazard ratio of 1.223 (95% CI: 1.089-1.373) (**Figure 2**). Zoster without complications presented 1.173-fold risk in TKR patients while comparing with non-TKR controls. However, most other secondary outcomes of herpes zoster did not present significance in future risk (**Figure 3**). The findings in HZ risk remained consistent across different covariate-matching models, wash-out periods and longer follow-up periods (**Table S2**).

### Stratified analyses by gender and age groups

The analysis was further stratified by gender and age groups to investigate potential moderators of the observed association between TKR and HZ risk. Compared to the control cohort, the hazard ratio of HZ was significantly higher for both females and older adults in the TKR cohort. As detailed in **Table 2**. the hazard ratio for males was 1.097 (95% CI: 0.887-1.357), while the hazard ratio for females was 1.146 (95% CI: 1.001-1.311). The hazard ratio of HZ for patients aged 65 years or older was significantly higher than non-TKR population in the same age subgroup (HR=1.195; 95% CI: 1.057-1.351). However, for people aged between 18-49 years old and 50-65 years old, the risk of HZ in TKR group was insignificant. In both age subgroups, female TKR patients presented statistically significant risk in developing HZ while comparing with non TKR female patients (50-64 years old, HR=1.543, 95% CI: 1.026-2.320; 65 years old above, HR=1.162, 95% CI: 1.008-1.339), whereas male TKR patients presented did not present significant risk in developing HZ while comparing with non TKR male patients (**Table 3**).

## Discussion

In this study, we investigated the association between TKR and the risk of HZ in OA patients. We found that TKR was associated with a higher risk of HZ compared to patients who did not undergo this procedure, and this association persisted across different follow-up periods up to 5 years and covariate-matching models. We also stratified different age and sex subgroups to discuss the potential role of the two covariates in the observed association.

The mechanisms behind the association between TKR and HZ are unclear, but they may involve multiple factors that impact the immune and nervous systems. HZ occurs when the varicella-zoster virus (VZV) reactivates, remaining dormant in the dorsal root ganglia following the initial infection^1^. Reactivation can be triggered by various factors, including immunosuppression, surgical stress, nerve injury, and exposure to VZV^19^-^22^. TKR is a major surgical intervention that can induce these risk factors, potentially leading to HZ development. First, TKR causes extensive tissue damage, blood loss, and inflammation, which may weaken the immune response and increase the susceptibility to HZ^23^. TKR also requires the use of perioperative medications, such as general anesthesia, opioids, blood transfusion, and immunosuppressive drugs^24^, ^25^, which may further suppress cell-mediated immunity and facilitate VZV reactivation. Furthermore, TKR-induced mechanical joint damage and somatosensory alterations may contribute to neural dysregulation and subsequent VZV reactivation within the dorsal root ganglia^26^-^28^. Additionally, the peri-operative environment during TKR presents potential exposures to VZV from healthcare personnel, visitors, or co-patients, particularly those lacking prior varicella-zoster vaccination. Therefore, TKR may increase the risk of HZ through a combination of immunological and neurological factors.

We also observed that the effect of TKR on HZ risk was modified by sex and age, suggesting some OA patients were more likely to develop HZ after TKR than others. This finding is in line with previous studies that identified age and sex as independent risk factors for HZ, with females and older adults having higher rates and worse outcomes of HZ than males and younger adults^29^. Several possible mechanisms may explain the effect modification by sex and age. First, older adults may have impaired cell-mediated immunity and increased inflammation, which make them more susceptible to HZ after TKR^29^, ^30^. Second, older adults may have more comorbid conditions, such as diabetes, hypertension, and cardiovascular diseases, that compromise their immune function and predispose them to HZ^29^. Third, females and older adults may respond differently to the factors associated with TKR, such as surgical stress, anesthesia, pain management, and recovery. For example, female and elderly patients usually require higher doses of opioids for pain relief, which suppress their cellular immunity and may increase their viral load of VZV^29^, ^31^, ^32^. Furthermore, female and elderly patients tend to have slower recovery and rehabilitation after TKR, which prolongs their exposure to potential triggers of HZ, such as nerve injury, inflammation, or infection^33^. Additionally, sex-specific hormonal and genetic factors may also contribute to the increased HZ risk observed in females after TKR. Fluctuating levels of female sex hormones, including estrogens and progesterone, are known to modulate immune responses to pathogens such as VZV^34^. Hormonal changes during reproductive life events like puberty, menstrual cycling, pregnancy, and menopause could potentially reactivate latent VZV^35^, ^36^. Additionally, genetic polymorphisms linked to altered viral immunity and latency have been identified; some of these high-risk alleles may occur at higher frequency in women^37^, ^38^. Further research is needed to elucidate the effects of female sex hormones and genetic susceptibility on HZ pathogenesis after major surgery like TKR.

It is important to note that TKR patients are susceptible to various other types of infections as well. Periprosthetic joint infection (PJI) is one of the most serious complications following TKR, with an incidence rate of approximately 1-2%^39^, ^40^. PJI can be caused by a variety of pathogens, with Staphylococcus aureus and coagulase-negative staphylococci being the most common^41^. Other less frequent but significant infections include surgical site infections, which can be superficial or deep, and occur in about 0.5-1.5% of TKR patients^42^. Urinary tract infections are also relatively common, particularly in the immediate postoperative period, with rates ranging from 1.5-4%^43^. Additionally, patients may develop pneumonia, especially those with prolonged hospital stays or those requiring mechanical ventilation^42^. These various infections not only impact patient outcomes and quality of life but also contribute significantly to healthcare costs and resource utilization^39^, ^40^. The risk factors for these infections often overlap with those for HZ, including advanced age, immunosuppression, and comorbidities such as diabetes^41^, ^42^. Therefore, a comprehensive approach to infection prevention and management is crucial in the care of TKR patients.

Our study has several strengths. First, we used a large sample size in each cohort, increasing our findings' statistical power and precision. Second, we utilized propensity score matching methods that enabled us to bring about similar baseline characteristics between the TKR and the control group thereby reducing confounding bias in our study. Third, we did sensitivity analyses and demonstrated the robustness of our findings.

However, our study also has some limitations that should be acknowledged. First, as this was an observational study, causal relationships between TKR and HZ cannot be definitively determined. Second, we used administrative data, which may have some inaccuracies or incompleteness in diagnosing and coding HZ and other variables. Third, we did not have information on some potential confounders, such as smoking status, body mass index, and family history of HZ. Fourth, since this study utilizes a U.S. database, the findings may not be fully representative of the broader population, limiting generalizability to other countries or healthcare systems with different demographics or clinical practices. Future studies should address these limitations, explore these issues further, and include more diverse populations to enhance the generalizability of the results. Fifth, the difference in subtypes of total knee replacement could potentially cause difference in the observed TKR-HZ association. In the current study, total knee replacement was identified based on ICD-10-PCS codes. In this case, we were not able to clearly identify whether the surgery was primary or revision type. Sixth, only a small portion of patients can be followed for 15 years, and this could reduce statistical power to produce robust results in the 15-year follow-up sensitivity analysis.

Despite these limitations, our study provides evidence that OA patients who undergo TKR have a higher risk of developing HZ infection, especially if they are female or older. This finding has profound clinical implications, as HZ can cause severe pain and complications. Clinicians caring for TKR patients should vigilantly monitor for signs and symptoms of HZ, including pain, tingling, itching, or rash, and promptly diagnose and initiate treatment if HZ is suspected. Certain high-risk subgroups, such as female and elderly TKR patients, may warrant particularly close surveillance for HZ in the postoperative period. Patients should be aware of the risks and benefits of TKR, including the possible increased risk of HZ, and discuss their risk factors and preventive measures, such as antiviral drugs or vaccines, with their physicians. Future research should explore the mechanisms and outcomes of HZ after TKR and develop risk prediction models that incorporate patient factors, such as age, gender, and comorbidities. This could help identify and prevent HZ in TKR patients and optimize prevention and treatment strategies.

In conclusion, this study demonstrated that TKR is associated with an increased risk of HZ, particularly for females and older adults. These findings highlight the importance of patient education regarding HZ risk after TKR, as well as the need for physicians to maintain a high index of suspicion for HZ in the post-TKR population. Further research is warranted to elucidate the mechanisms underlying the increased HZ incidence post-TKR and identify optimal preventive, diagnostic, and therapeutic strategies to mitigate HZ burden in these patients. Implementing targeted education, prevention, diagnosis, and management protocols may improve outcomes and reduce complications in TKR patients at risk for HZ reactivation.

## Supplementary Material

Supplementary tables.

## Figures and Tables

**Table 1:** Baseline characteristics of study subjects (before and after propensity score matching)

	Before matching				After matching^a^		
	TKR cohort (n = 43,670)	Control cohort (n = 2,073,676)	Standardized difference		TKR cohort (n = 43,670)	Control cohort (n=43,670)	Standardized difference
**Age at index**							
Mean±SD	64.3 ± 10.4	60.6 ± 14.6	**0.29**		64.3 ± 10.4	64.3 ± 10.8	0.01
**Sex**							
Male	16851 (38.6)	805087 (38.8)	0.00		16851 (38.6)	16914 (38.7)	0.00
Female	26723 (61.2)	1213427 (58.5)	0.05		26723 (61.2)	26662 (61.1)	0.00
**Race, n (%)**							
White	31364 (71.8)	1404380 (67.7)	0.09		31364 (71.8)	31389 (71.9)	0.00
Black or African American	4741 (10.9)	259381 (12.5)	0.05		4741 (10.9)	4719 (10.8)	0.00
Asian	1267 (2.9)	45289 (2.2)	0.05		1267 (2.9)	1267 (2.9)	0.00
Native Hawaiian or other Pacific Islander	388 (0.9)	7665 (0.4)	0.07		388 (0.9)	404 (0.9)	0.00
**Lifestyle**							
Alcohol dependence, smoking and substance use	2392 (5.5)	128739 (6.2)	0.03		2392 (5.5)	2179 (5.0)	0.02
**Comorbidities**							
Hypertension	12263 (28.1)	456075 (22.0)	**0.14**		12263 (28.1)	12052 (27.6)	0.01
Diabetes mellitus	4506 (10.3)	197939 (9.5)	0.03		4506 (10.3)	4406 (10.1)	0.01
Hyperlipidemia	7098 (16.3)	267686 (12.9)	0.09		7098 (16.3)	6908 (15.8)	0.01
Ischemic heart diseases	3104 (7.1)	122550 (5.9)	0.05		3104 (7.1)	2901 (6.6)	0.02
Chronic kidney disease	1120 (2.6)	54785 (2.6)	0.00		1120 (2.6)	923 (2.1)	0.03
Chronic obstructive pulmonary disease	1163 (2.7)	56763 (2.7)	0.00		1163 (2.7)	991 (2.3)	0.03
Ankylosing spondylitis	52 (0.1)	2191 (0.1)	0.00		52 (0.1)	27 (0.1)	0.02
Systemic lupus erythematosus	158 (0.4)	9752 (0.5)	0.02		158 (0.4)	92 (0.2)	0.03
Sjögren syndrome	133 (0.3)	6284 (0.3)	0.00		133 (0.3)	86 (0.2)	0.02
Psoriasis	302 (0.7)	14742 (0.7)	0.00		302 (0.7)	218 (0.5)	0.03
**Socioeconomic Status**							
Health hazards related to socioeconomic and psychosocial circumstances	214 (0.5)	14202 (0.7)	0.03		214 (0.5)	147 (0.3)	0.02
**Medical Utilization Status**							
Ambulatory visit	27839 (63.7)	1128558 (54.4)	**0.19**		27839 (63.7)	27711 (63.5)	0.01
Inpatient visit	10930 (25.0)	288753 (13.9)	**0.28**		10930 (25.0)	10778 (24.7)	0.01
**Vaccination Status**							
Encounter for herpes zoster vaccination	3884 (8.9)	169650 (8.2)	0.03		3884 (8.9)	3831 (8.8)	0.00
**Laboratory data**							
BMI, n (%)							
≥ 25 (kg/m^2^)	8572 (19.6)	259197 (12.5)	**0.20**		8572 (19.6)	8576 (19.6)	0.00
C reactive protein, n (%)							
≥ 3 (mg/L)	3434 (7.9)	73956 (3.6)	**0.19**		3434 (7.9)	3169 (7.3)	0.02
Procalcitonin, n (%)							
≥ 10 (ng/ml)	10 (0.0)	146 (0.0)	0.01		10 (0.0)	10 (0.0)	0.00

Bold font represents a standardized difference was more than 0.1; In order to protect privacy of individuals, in the TriNetX research network, if the patient amount is less or equal to 10, results show the count as 10 ^a^ Propensity score matching was performed on age at index, sex, race, body mass index, lab data, status of comorbidities, lifestyle, socioeconomic issues, medical utilization status, vaccination for herpes zoster status.

**Table 2:** Stratification analysis of herpes zoster in total knee replacement patients

	Cases occurring new-onset herpes zoster		
Subgroups	Total knee replacement cohort (No. of event/ Total knee replacement patient amount in each subgroup)	Control cohort (No. of event/ non-total knee replacement patient amount in each subgroup)	HR (95% CI)^a,b^
**Gender**			
Male	181/16851	160/16851	1.097 (0.887,1.357)
Female	461/26721	392/26721	**1.146 (1.001,1.311)**
**Age at index date**			
18-49 years old^c^	≤ 10/373	≤ 10/373	0.463 (0.116,1.853)
50-64 years old	78/6149	62/6149	1.245 (0.892,1.737)
≥ 65 years old	592/38492	486/38492	**1.187 (1.052,1.338)**

^a^ Propensity score matching was performed on age at index, sex, race, body mass index, lab data, status of comorbidities, lifestyle, socioeconomic issues, medical utilization status, vaccination for herpes zoster status. ^b^ Follow-up time in all analyses in this table was set as 5-years, starting from 90 days after index date ^c^ In order to protect privacy of individuals, in the TriNetX research network, if the patient amount is less or equal to 10, results show the count as 10

**Table 3:** Herpes zoster risk stratified by sex in different age subgroups.

	Cases occurring new-onset herpes zoster		
Subgroups	Total knee replacement cohort (No. of event/ Total knee replacement patient amount in each subgroup)	Control cohort (No. of event/ non-total knee replacement patient amount in each subgroup)	HR (95% CI)^a,b^
**50-64 years old**			
Male	19/2517	23/2517	0.829 (0.452,1.522)
Female	59/3590	38/3590	**1.543 (1.026,2.320)**
**≥ 65 years old**			
Male	165/14699	144/14699	1.111 (0.889,1.389)
Female	419/23559	352/23559	**1.162 (1.008,1.339)**

^a^ Propensity score matching was performed on age at index, sex, race, body mass index, lab data, status of comorbidities, lifestyle, socioeconomic issues, medical utilization status, vaccination for herpes zoster status. ^b^ Follow-up time in all analyses in this table was set as 5-years, starting from 90 days after index date
