# Supplementary material for: Herpes Zoster Risk After Total Knee Replacement: a multicenter, propensity-score-matched cohort study in the United States
Source: Int J Med Sci. 2024 Aug 19;21(12):2244–51. doi: 10.7150/ijms.97654 (PMC11413905; doi:10.7150/ijms.97654)
Supplement: Supplementary file 1 — Supplementary tables. [file ijmsv21p2244s1.pdf]

Supplementary Files

Supplementary Tables

Table S1. Utilized definitions of study population, covariates and outcome events

**Description**	**Adopted codes** [a]
Study Population	
Osteoarthritis	ICD-10-CM: M15-M19
Procedures	
Total knee replacement	ICD-10-PCS[b] : 0SRD0JZ, 0SRC0JZ, 0SRW0JZ, 0SRV0JZ, 0SRU0JZ, 0SRT0JZ, 0SRD07Z, 0SRD0KZ, 0SRU07Z, 0SRU0KZ, 0SRW07Z, 0SRW0KZ, 0SRC0KZ, 0SRT07Z, 0SRT0KZ, 0SRV0KZ
Confounding factors and comorbidities	
Neoplasms	ICD-10-CM: C00-D49
Diabetes mellitus	ICD-10-CM: E08-E13
Hypertension	ICD-10-CM: I10
Ischemic heart diseases	ICD-10-CM: I20-I25
Hyperlipidemia	ICD-10-CM: E78.5
Chronic kidney disease	ICD-10-CM: N18
Chronic obstructive pulmonary disease	ICD-10-CM: J44
Ankylosing spondylitis	ICD-10-CM: M45
Systemic lupus erythematosus	ICD-10-CM: M32
Sjögren syndrome	ICD-10-CM: M35.0
Psoriasis	ICD-10-CM: L40

Health hazards related to socioeconomic and psychosocial circumstances	ICD-10-CM: Z55-Z65
Substance abuse	ICD-10-CM: F10-F19
Encounter for herpes zoster vaccination	ICD-10-CM: Z23
Encounter for herpes zoster vaccination: Zostavax® Merck, Shingrix GSK.	CPT code: 90736, 90750
Medications	
Glucocorticoids	VA code: HS051
Opioid analgesics	VA code: CN101
Diclofenac	RxNorm: 140587
Celebrex	RxNorm: 3355
Outcomes	
Herpes zoster	B02
Zoster encephalitis	B02.0
Zoster with other nervous systemic involvement	B02.2
Zoster ocular disease	B02.3
Disseminated zoster	B02.7
Zoster with other complications	B02.8
Zoster without complications	B02.9

[a]ICD-10-CM: International Classification of Diseases, Tenth Revision, Clinical Modification

[b]ICD-10-PCS: International Classification of Diseases, Tenth Revision, Procedure Coding System

Table S2. Sensitivity analyses: hazard ratio of herpes zoster with 95% confidence interval under various conditions.

**Various matching covariates**	**Model 1[a]**	**Model 2[b]**	**Model 3[c]**	**Model 4[d]**
Non-TKR controls	1.00	1.00	1.00	1.00
TKR patients	**1.219(1.085,1.369)**	**1.173(1.046,1.315)**	**1.127(1.006,1.263)**	**1.215 (1.126,1.312)**
**Various wash-out periods**	**Model 1[e]**	**Model 2[e]**	**Model 3[f]**	
Non-TKR controls	1.00	1.00	1.00	
TKR patients	**1.200(1.060,1.359)**	**1.197(1.04,1.377)**	**1.256(1.061,1.487)**	
**Various follow-up times**	**Model 1[g]**	**Model 2[h]**	**Model 3[i]**	
Non-TKR controls	1.00	1.00	1.00	
TKR patients	**1.195(1.077,1.325)**	**1.144(1.038,1.261)**	**1.139(1.037,1.25)**	

[a] Propensity score matching was performed on age at index, sex, race and comorbidities.

[b] Propensity score matching was performed on age at index, sex, race, body mass index, lab data, status of comorbidities, lifestyle, socioeconomic issues, medical utilization status, vaccination for herpes zoster status and comedication status.

[c] Propensity score matching was performed on age at index, sex, race, body mass index, lab data, status of comorbidities, lifestyle, socioeconomic issues, medical utilization status, vaccination for herpes zoster status in different definition (Zostavax® Merck, Shingrix GSK.; CPT code: 90736, 90750).

[d] Crude hazard ratio that did not undergo propensity score matching

[d] Wash-out period was set as 12 months in this model. Incident herpes zoster occurred within 12 months were not calculated as outcome events.

[e] Wash-out period was set as 24 months in this model. Incident herpes zoster occurred within 24 months were not calculated as outcome events.

[f] Wash-out period was set as 36 months in this model. Incident herpes zoster occurred within 36 months were not calculated as outcome events.

[g] Follow-up period was set as 8 years in this model.

[h] Follow-up period was set as 10 years in this model.

[i] Follow-up period was set as 15 years in this model.
